# Hepatitis C virus NS3 protein enhances hepatocellular carcinoma cell invasion by promoting PPM1A ubiquitination and degradation

**DOI:** 10.1186/s13046-017-0510-8

**Published:** 2017-03-10

**Authors:** Yali Zhou, Yan Zhao, Yaoying Gao, Wenjun Hu, Yan Qu, Ning Lou, Ying Zhu, Xiaoping Zhang, Hongmei Yang

**Affiliations:** 1 Department of Pathogenic Biology, School of Basic Medicine, Tongji Medical College, Huazhong University of Science and Technology, Wuhan, 430030 Hubei Province China; 2 Department of Urology, Union Hospital, Tongji Medical College, Huazhong University of Science and Technology, Wuhan, 430022 Hubei Province China; 3 State Key Laboratory of Virology and College of Life Sciences, Wuhan University, Wuhan, 430072 Hubei Province China

**Keywords:** Hepatocellular carcinoma, Hepatitis C virus, PPM1A, Cancer cell invasion, Ubiquitination and degradation

## Abstract

**Background:**

Growing evidence suggests that hepatitis C virus (HCV) contributes to hepatocellular carcinoma (HCC) by directly modulating oncogenic signaling pathways. Protein phosphatase magnesium-dependent 1A (PPM1A) has recently emerged as an important tumor suppressor as it can block a range of tumor-centric signaling pathways through protein dephosphorylation. However, the role and regulatory mechanisms of PPM1A in HCV-infected cells have not been reported.

**Methods:**

Total, cytoplasmic, and nuclear PPM1A protein after HCV infection or overexpression of HCV nonstructural protein 3 (NS3) were detected by western blotting. The expression of PPM1A in normal liver and HCV-related HCC tissues was quantified by immunohistochemistry. The effects of HCV infection and NS3 expression on the PPM1A protein level were systematically analyzed, and the ubiquitination level of PPM1A was determined by precipitation with anti-PPM1A and immunoblotting with either anti-ubiquitin or anti-PPM1A antibody. Finally, the roles of NS3 and PPM1A in hepatoma cell migration and invasion were assessed by wound healing and transwell assays, respectively.

**Results:**

HCV infection and replication decreased PPM1A abundance, mediated by NS3, in hepatoma cells. Compared to normal liver tissues, the expression of PPM1A was significantly decreased in the HCC tumor tissues and adjacent non-tumor tissues. NS3 directly interacted with PPM1A to promote PPM1A ubiquitination and degradation, which was dependent on its protease domain. Blockade of PPM1A through small interfering RNA significantly promoted HCC cell migration, invasion, and epithelial mesenchymal transition (EMT), which were further intensified by TGF-β1 stimulation, in vitro. Furthermore, restoration of PPM1A abrogated the NS3-mediated promotion of HCC migration and invasion to a great extent, which was dependent on its protein phosphatase function.

**Conclusions:**

Our findings demonstrate that the HCV protein NS3 can downregulate PPM1A by promoting its ubiquitination and proteasomal degradation, which might contribute to the migration and invasion of hepatoma cells and may represent a new strategy of HCV in carcinogenesis.

**Electronic supplementary material:**

The online version of this article (doi:10.1186/s13046-017-0510-8) contains supplementary material, which is available to authorized users.

## Background

Hepatocellular carcinoma (HCC) is one of the most common cancers worldwide, particularly in China [1]. It ranks as the fifth most common malignancy and the second leading cause of cancer deaths worldwide, with more than 695,900 deaths each year. Chronic hepatitis C virus (HCV) infection is one of the most important risk factors for developing HCC [2]. Approximately 200 million people are infected with HCV worldwide, and up to 80% of infected individuals progress to chronic hepatitis, which results in liver cirrhosis and HCC in many cases [3, 4]. Although some progress has been made, the mechanism underlying HCV-associated hepatocarcinogenesis remains not fully understood.

HCV is a positive single-stranded RNA virus with an exclusively cytoplasmic life cycle. Unlike hepatitis B virus (HBV), which is a DNA virus that can induce insertional mutagenesis, HCV does not insert into the host cell genome [5]. Although the inflammation caused by chronic hepatitis C is likely to contribute to the development of HCC, there is strong evidence that one or more of the viral proteins and its involvement in interrupting cellular signaling pathways contribute mostly to tumorigenesis [6]. Multiple cellular signaling pathways including the Wnt/β-catenin, p53, pRb, MAPK pathways, and particularly, the TGF-β/smad pathway, have been implicated in hepatocarcinogenesis [6–8]. TGF-β appears to play an important role in the pathogenesis of HCC and is considered a hallmark of HCC because it is elevated in the serum, tissue, and urine of patients and the increased levels correlate with tumor progression and survival [9–11]. Furthermore, TGF-β can induce a protumoral transcriptional program in cells that express HCV subgenomic replicon [12]. Moreover, previous studies have shown that blockade of the TGF-β signaling pathway using inhibitors dramatically suppresses HCC cell invasiveness and metastasis [13]. As these signaling pathways are all associated with the development of HCC, targeting and/or blocking of these pathways can be expected to contribute to new strategies for suppressing tumorigenesis and disease progression.

PPM1A, also known as PP2Cα, is a protein phosphatase that belongs to the Protein Phosphatase 2C family. PPM1A has recently emerged as an important tumor suppressor owing to its involvement in the regulation of several tumor-centric signaling pathways, including TGF-β/smad [14], Wnt/β-catenin [15], MAPK [16], PI3K/Akt [17], and NF-κB [18, 19]. The regulation of these pathways has been attributed to PPM1A phosphatase activity towards important pathway components (e.g., p-Smad2/3 in TGF-β, Axin in Wnt, p38 in MAPK, p85 subunit of PI3K in PI3K, and RelA and IKKβ in NF-κB). Moreover, overexpression of PPM1A led to cell cycle arrest in G2/M and apoptosis by inducing both the expression and transcriptional activity of p53, although it remains unclear how PPM1A modulates p53 activity [20]. Importantly, several tumor tissues, including metastatic human prostate and bladder carcinomas, have shown decreased or loss of PPM1A expression [18, 21]. This indicates that modulation of the protein level of PPM1A might be an effective strategy for the abnormal activation of the signal pathway in tumor progression. However, the expressional regulation and distinct functions of PPM1A in regulating tumor cells remain largely unknown.

In this study, we examined the expression of PPM1A in HCV-infected hepatoma cells and HCV-related HCC tissues, and we determined whether this protein is involved in HCV-related HCC development. We found a direct link between HCV infection and cellular abundance of PPM1A in both hepatoma cells and the HCC tissues. The mechanism by which NS3 downregulates PPM1A abundance was revealed, and the roles of PPM1A in regulating hepatoma cell invasion and migration were assessed in vitro. Together, our findings provide novel evidence on the mechanisms involved in HCV-mediated progression of HCC, which may provide potential candidates for the clinical prevention and treatment of HCV-associated HCC.

## Methods

### Cell culture and virus preparation

Human HCC cells (Huh-7 and Huh-7.5.1) and embryo kidney cells (HEK293T) were purchased from the China Center for Typical Culture Collection (Wuhan, China). Cells were maintained in Dulbecco’s modified Eagle’s medium (DMEM) supplemented with 10% fetal bovine serum (FBS) and 1% penicillin and streptomycin in an incubator at 37 °C with 5% CO_2_. The genotype 2a HCV strain JFH1 was kindly gifted by Dr. Ying Zhu. Three days after infection of Huh-7.5.1 cells with JFH1, cell culture supernatant containing HCV particles (HCVcc) was collected, filtered using a 0.45-μm filter, and stored at −80 °C for later infection of Huh-7 cells. HCV titers were measured using a HCV RNA qPCR Diagnostic Kit (KHB Co., Shanghai, China).

### Reagents and antibodies

Protein synthesis inhibitor cycloheximide (CHX, 300 μM), proteasome inhibitor MG132 (20 μM), and autophagy inhibitor chloroquine (diphosphate salt, 50 μM) were purchased from Sigma-Aldrich (St. Louis, MO, USA). All inhibitors were dissolved in DMSO and used at the final concentrations indicated above. Recombinant Human TGF-β1 was purchased from PeproTech (Rocky Hill, NJ, USA), dissolved in citric acid buffer (10 mM, pH 3.0) at a concentration of 1 mg/ml, and used at a final concentration of 200 pM.

Mouse monoclonal antibodies against HCV NS3 and core protein were obtained from Abcam (Cambridge, MA, USA) and Thermo Fisher Scientific (Waltham, MA, USA), respectively. Rabbit polyclonal antibody against HCV NS5A was purchased from ViroGen (Watertown, MA, USA). Antibodies against PPM1A, E-cadherin, N-cadherin, and vimentin were purchased from Cell Signaling Technology (Danvers, MA, USA), and antibodies against Flag and GFP were obtained from Sigma-Aldrich (St. Louis, MO, USA).

### Clinical specimens and immunohistochemistry

Twelve pairs of HCV-related HCC tumor tissues and matched adjacent non-tumor tissues, and 10 normal liver tissue samples were collected at the Department of Pathology, Union Hospital, Tongji Medical College of Huazhong University of Science and Technology, between 2015 and 2016. The study was approved by the ethics committee of Tongji Medical College, and informed consent was obtained from all participants. Immunohistochemical staining analyses were performed using 4-μm formalin-fixed paraffin-embedded tissue sections. The sections were deparaffinized, rehydrated, and incubated in EDTA at 120 °C for 5 min for antigen retrieval. After incubation with 3% H_2_O_2_ at room temperature for 15 min, the sections were blocked with fetal bovine serum and incubated with primary antibodies overnight. Immunodetection was performed with HRP-conjugated goat anti-rabbit antibody. Finally, the immune complexes were visualized with a chromogenic substrate (DAB; DAKO, Glostrup, Denmark), and the sections were counterstained with hematoxylin.

The score of PPM1A staining was determined and evaluated as described by Soslow RA et al. [22]. Briefly, the staining intensity was evaluated independently by two experienced pathologists and was given a score from 0 to 3 (0 = no, 1 = weak, 2 = moderate, 3 = strong staining). The intensity score was multiplied by the proportion of cells stained (%) to give a final score.

### Plasmids and transfection

Flag-PPM1A (pRK5F) and Flag-PPM1A D239N (pRK5F), the phosphatase-dead mutant of PPM1A, were provided as a gift by Dr. Xinhua Feng [14]. Flag-tagged expression plasmids for HCV core, P7, E1, E2, NS2, NS3, NS4A, NS4B, NS5A, and NS5B proteins (pCMV-tag2B) were generously gifted by Dr. Ying Zhu. To construct the GFP-tagged NS3 and NS3 deletion constructs (protease and helicase domains of NS3), corresponding sequences were amplified by PCR using Flag-NS3 as the template and were subcloned into the pGFP vector (Clontech Laboratories Inc., Mountain View, CA, USA) to derive pGFP-NS3, pGFP-protease, and pGFP-helicase. Site-directed mutagenesis was carried out according to the manufacturer’s instructions (TransGen Biotech, Beijing, China) to convert the serine codon at position 139 of wild-type NS3 to an alanine codon to create a protease-inactive mutant of NS3, called S139A. All constructs containing PCR fragments were confirmed by DNA sequencing.

Cells were grown to 50% confluence in 6-well plates, and transfections were conducted with Lipofectamine 2000 (Invitrogen, Carlsbad, CA, USA), according to the manufacturer’s instructions. In all co-transfection experiments, corresponding vectors were used as negative controls to ensure similar DNA concentrations. Cells were either used at 24 h post-transfection for wound healing and invasion assay, or at 36 h for western blotting.

### RNA interference analysis

Short interfering RNA (siRNA) against PPM1A (si-PPM1A) and negative control (si-NC) with nonspecific targeting sequences were synthesized by GenePharma Co. (Shanghai, China). The sequences were as follows:

PPM1A siRNA 1 (5′-GTACCTGGAATGCAGAGTA-3′); PPM1A siRNA 2 (5′-GTCGACACCTGTTTGTATA-3′); NC (non-targeting) siRNA (5′-TTCTCCGAACGTGTCACGT-3′). Huh-7 cells were grown to 40% confluence in 6-well plates and transiently transfected with 100 nM siRNAs using Lipofectamine 2000. The cells were used either for in vitro wound healing and invasion assays after 24 h of transfection or for western blotting after 36 h.

### RNA isolation and quantitative reverse transcription (qRT-)PCR

Total RNA was extracted from cells using Trizol reagent (Invitrogen) and mRNA was reverse transcribed using a Revert Aid First-Strand cDNA Synthesis Kit (Thermo Fisher) according to the manufacturer’s instructions. qPCR was carried out with 2× SYBR Green Mix (Thermo Fisher) on a LightCycler 480II (Roche). The primers used in real-time PCR were as follows:

GAPDH: (F: 5′-GGTGAAGGTCGGAGTCAACGG-3′; R: 5′-GAGGTCAATGAAGGGGTCATTG-3′), PPM1A: (F: 5′-CGCTGGAGAAAGAACGAAT-3′; R: 5′-TCTCTATCTGCCCACAGCCTAC-3′). HCV: (F: 5′-TCTGCGGAACCGGTGAGTA-3′; R: 5′-TCAGGCAGTACCACAAGGC-3′). The data were normalized to that of glyceraldehyde-3-phosphate dehydrogenase (GAPDH). Relative expression was calculated using the 2^-ΔΔCt^ method.

### Western blotting

Total protein was extracted using RIPA protein lysis buffer (Beyotime, Shanghai, China) with freshly added 1% protease inhibitor cocktail and 1 mM phenylmethylsulfonyl fluoride (PMSF). Cell fractions were prepared using a Nuclear and Cytoplasmic Protein Extraction Kit (Beyotime) according to the manufacturer’s protocol. In total, 50 μg of protein was used for western blotting. Samples were separated by SDS-PAGE and transferred onto PVDF membranes. After blocking in 5% skim milk, the PVDF blots were incubated with primary antibodies in blocking buffer overnight at 4 °C and then with HRP-conjugated secondary antibody for 2 h. Reactive bands were visualized with ECL reagent (Pierce, Rockford, IL) and analyzed. Protein expression was quantified using ImageJ software.

### Co-immunoprecipitation

Cells were washed twice with ice-cold PBS and lysed in Triton-lysis buffer [20 mM Tris-HCl (pH 7.5), 150 mM NaCl, 5 mM EDTA, 2 mM dithiothreitol (DTT), 0.5% Triton X-100, 1% protease inhibitor cocktail, and 1 mM PMSF]. The lysates were centrifuged at 12,000 × *g* for 10 min at 4 °C and supernatant was precleared with 20 μL Protein A/G PLUS-Agarose (Santa Cruz, CA, USA) for 1 h at 4 °C. The lysates were incubated with the appropriate antibody overnight at 4 °C, followed by precipitation with protein A/G PLUS-Agarose. The immunoprecipitates were collected by washing and centrifugation for three times, boiled in 2× SDS sample buffer, and subjected to western blotting.

### Immunofluorescence staining

Cells grown on coverslips were washed twice with ice-cold PBS, fixed in 4% paraformaldehyde, permeabilized with 0.3% Triton X-100 for 10 min, and blocked with 3% bovine serum albumin. Then, the cells were incubated with primary antibodies, followed by Alexa Fluor 488- or Alexa Fluor 594-conjugated secondary antibody (Molecular Probes, OR, USA). Nuclei were stained with DAPI.

### In vitro invasion assay

Twenty-four-well transwell plates with 8-μm pore-size polycarbonate membrane inserts (Corning, NY, USA) were precoated with 80 μL of 1:8 DMEM-diluted Matrigel (BD Biosciences, CA, USA). Cells (5 × 10^4^) were seeded in serum-free medium in the top chamber and allowed to invade into the lower chamber, which contained 20% FBS as a chemoattractant. TGF-β1 or vehicle was added to the upper and lower chambers. After 24 h, cells that had invaded into the lower surface of the membrane were fixed in 100% methanol, stained with 0.1% crystal violet, and quantified by counting in five random fields.

### In vitro wound healing assay

Cells grown to confluence in 24-well transwell plates were manually scratched with a micropipette tip to create uniformly sized wounds. Then, the cell culture medium was replaced with fresh FBS-free medium, and TGF-β1 or vehicle was added as required. Four points were randomly selected and marked for each scratch, and healing wounds were imaged at 36 h. The percentage of wound closure was calculated based on the initial measurement for that point at time point zero.

### Statistical analysis

All values are presented as the mean ± standard error (SEM) from at least three independent experiments. Differences between group means were determined using a two-tailed Student’s *t*-test or one-way ANOVA. A *P*-value <0.05 was considered statistically significant.

## Results

### HCV infection downregulates PPM1A abundance

**Fig. 1:**
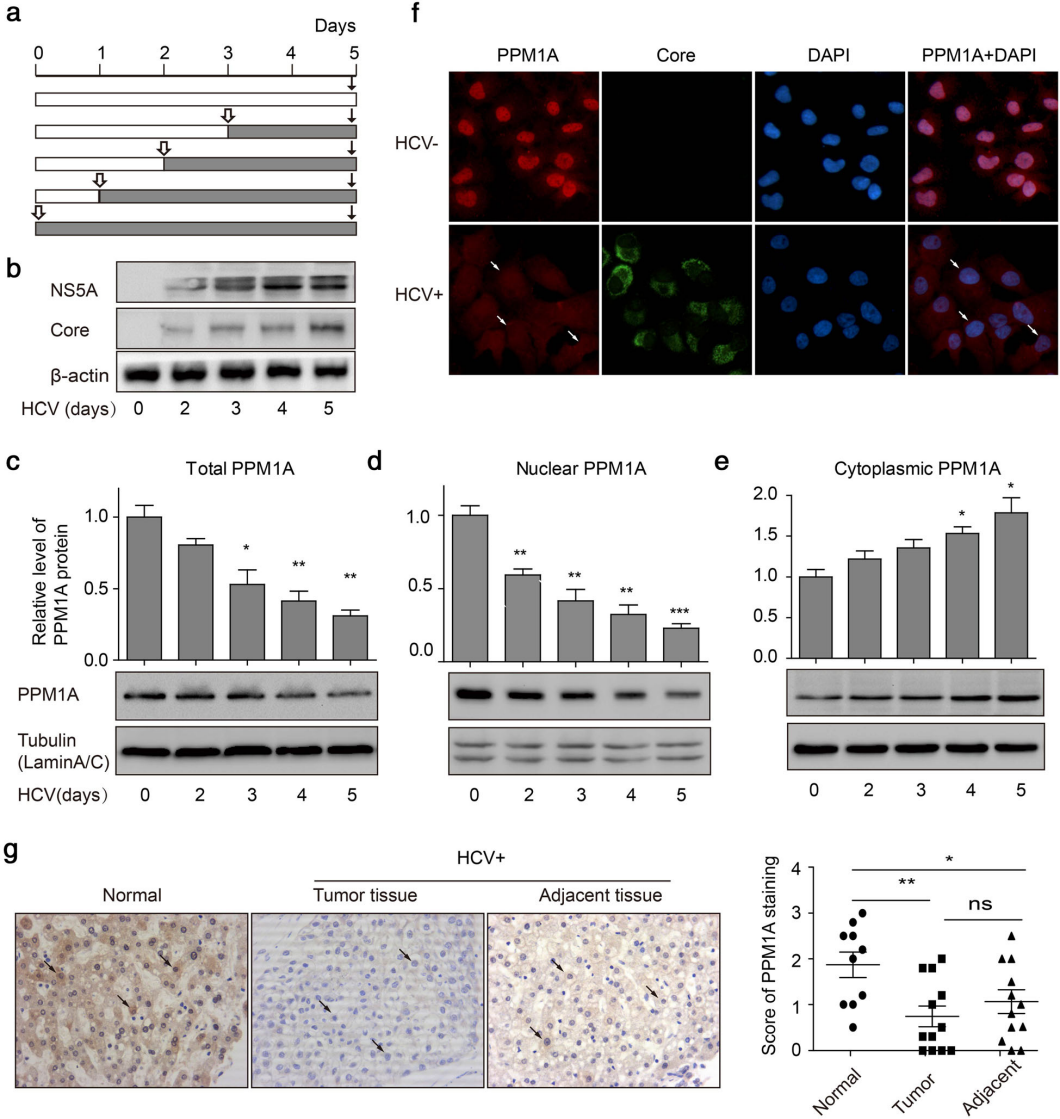
PPM1A abundance is negatively regulated in HCV-infected cells. **a** Schematic representation of the experiments shown in (**b**–**e**). Huh-7 cells were infected with JFH1 virus for 0–5 days at 1 multiplicity of infection (MOI) at intervals, and subsequently lysed simultaneously for immunoblot analysis. *Hollow bars* show uninfected cells; *shaded bars*, infected cells. **b** Levels of HCV infection in Huh-7 cells were determined by immunoblotting for HCV NS5A and core protein as described in a. **c**–**e** Total (**c**), nuclear (**d**), and cytoplasmic (**e**) PPM1A protein levels were measured by western blotting. The *bar graph* (mean ± SEM) displays protein quantification (*n* = 3). Data are normalized to a loading control (tubulin for total and cytoplasmic PPM1A, and lamin A/C for nuclear PPM1A) and expressed as the fold change relative to the protein levels in uninfected cells. **f** Cells were infected or not with JFH1 for 3 days, and the subcellular localization of PPM1A was monitored by dual immunolabeling of HCV core protein (*green*) and PPM1A (*red*). *Arrows* denote HCV-infected cells in which PPM1A expression and subcellular localization are significantly changed. **g** Immunohistochemistry was used to measure the expression of PPM1A (*brown staining*) in normal liver tissues (*n* = 10), and HCV-related HCC tissues and adjacent tissues (*n* = 12 each). g Immunohistochemistry was used to measure the expression of PPM1A (*brown staining*) in normal liver tissues (*n* = 10), and HCV-related HCC tissues and adjacent tissues (*n* = 12 each). *Arrows* indicate representative staining of PPM1A. *Left* panels show representative images of PPM1A expression, quantitative data are shown in the *right* panel. Data are the mean ± SEM. *P < 0.05, **P < 0.01 and ***P < 0.001 as evaluated using Student’s *t*-test or one-way ANOVA

To investigate the effect of HCV infection on PPM1A abundance, a genotype 2a virus strain, JFH1, was used to infect the human hepatic cell line Huh-7, which is highly permissive for HCV replication. HCV infection levels were quantified at both protein and RNA levels with the employment of immunoblotting and RT-qPCR analyses, respectively (Fig. 1b and Additional file 1: Figure S1). The results showed that PPM1A abundance was significantly reduced within 2–3 days of infection with JFH1 (Fig. 1c), and the PPM1A level at 5 days after infection was approximately 30% that of uninfected cells as indicated by the immunoblots. Cells were further separated into nuclear and cytoplasmic fractions. As shown in Fig. 1d and e, nuclear expression of PPM1A decreased more dramatically than total PPM1A, while the cytoplasmic abundance slightly increased during this period. Immunofluorescence analysis confirmed the strikingly lower abundance of PPM1A in HCV-infected cells. In mock-infected cells, PPM1A was mainly localized in the nucleus. In contrast, in HCV-infected cells, PPM1A showed a striking relocalization to the cytoplasm, in which HCV replicated and completed its life cycle (Fig. 1f).

To further examine how HCV affects the expression of PPM1A in HCC, we used immunohistochemistry to assess the expression levels of PPM1A in HCV-related HCC and normal liver tissues. As shown in Fig. 1g, compared with the normal liver tissues, significantly lower levels of PPM1A were found in the HCC tumor and adjacent tissues. In addition, there were no significant differences in PPM1A expression between tumor tissues and the paired adjacent non-tumor tissues. Together, these results provided strong evidence that PPM1A is downregulated in HCV-infected hepatoma cells.

### NS3 protein mediates the regulation of PPM1A

**Fig. 2:**
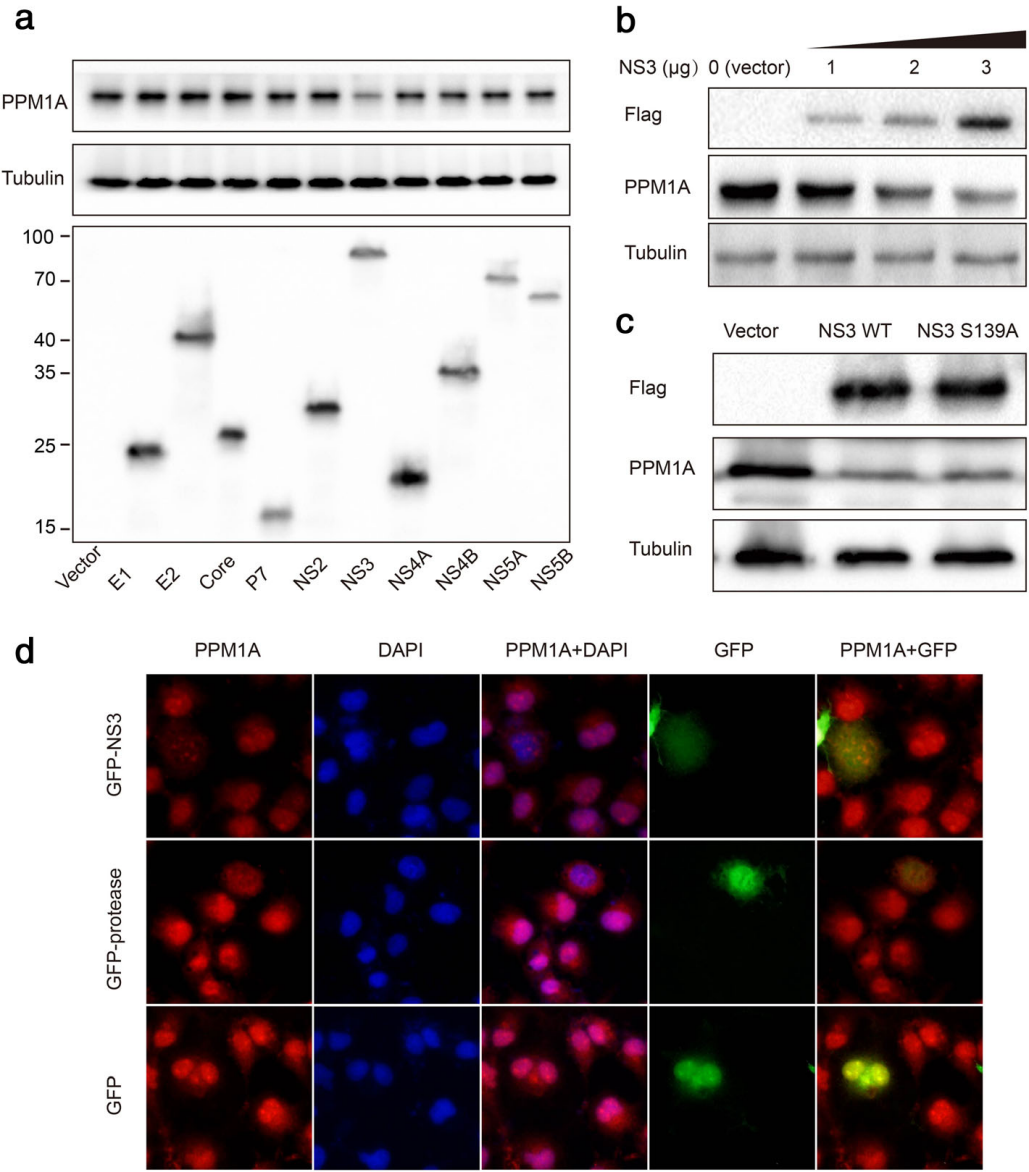
NS3 is responsible for the regulation of PPM1A abundance. **a**–**c** Huh-7 cells were transiently transfected with plasmid encoding one of the indicated HCV proteins (**a**), Flag-vector or Flag-NS3 plasmids at different concentrations as indicated (**b**), and NS3 or NS3 S139A plasmids (**c**) for 48 h and subsequently collected for western blotting. **d** Huh-7 cells were transfected with GFP-vector, GFP-NS3 or GFP-protease for 48 h and subsequently stained for PPM1A (*red*); the intracellular localization of PPM1A was examined by fluorescence microscopy

We hypothesized that the reduction in PPM1A abundance might be mediated by one or more viral proteins expressed during infection. Thus, we constructed a panel of expression plasmids involving all 10 HCV proteins with N-terminal epitope tags and transfected these into Huh-7 cells to screen for their ability to downregulate PPM1A expression. Transient transfection of NS3, but not other HCV proteins, significantly reduced PPM1A expression (Fig. 2a). A previous study on the virus-host protein–protein interaction network during HCV infection recently reported that NS3 protein could interact with PPM1A [22]. To confirm the effect of NS3 on PPM1A abundance in this model, Huh-7 cells were transfected with increasing doses of NS3. As shown in Fig. 2b, NS3 downregulated PPM1A in a dose-dependent manner.

NS3 is a serine protease that cleaves its substrates [23]. It is possible that the reduction of PPM1A is caused by its protease activity. Thus, a protease-inactive mutant of NS3, S139A, was constructed [24]. S139A had the same effect as the wild type on the expression of PPM1A (Fig. 2c), indicating that the protease activity is not necessary for the effect of NS3 on PPM1A expression.

Full-length NS3 and the NS3 protease domain are diffusely distributed in the cytoplasm and nucleus in the absence of the cofactor NS4A, while in the presence of NS4A, NS3 is localized exclusively in the cytoplasm [25]. The current study confirmed the cytoplasmic and nuclear distribution of NS3. Furthermore, it was shown that in GFP- and non-transfected cells, PPM1A was localized mainly in the nucleus. In contrast, in NS3-transfected cells, PPM1A demonstrated a more diffuse pattern throughout the cytoplasm and the nucleus, and total fluorescence intensity was strikingly reduced (Fig. 2d). Collectively, these results suggested that the HCV protein NS3 is sufficient to modulate the abundance and cellular localization of PPM1A.

### NS3 interacts with PPM1A

**Fig. 3:**
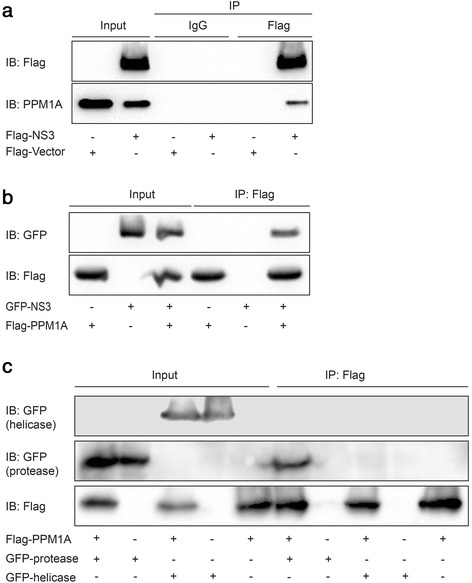
NS3 protein interacts with PPM1A. **a**–**c** Huh-7 cells were transiently transfected with Flag-NS3 or vector (**a**); HEK293T cells were cotransfected with Flag-PPM1A and GFP-NS3 (**b**). HEK293T cells were cotransfected with Flag-PPM1A and GFP-protease or GFP-helicase (**c**), 36 h later, co-IP was performed to identify the interaction between PPM1A and NS3 or its deletion mutants

It was hypothesized that NS3 might downregulate PPM1A through its interaction with PPM1A. Hence, we first examined whether NS3 interacts with endogenous PPM1A in Huh-7 cells. Huh-7 cells were transfected with Flag-NS3 plasmid and co-immunoprecipitation experiments were performed, which indicated that NS3 interacts with endogenous PPM1A in Huh-7 cells (Fig. 3a). In addition, we co-transfected HEK 293 T cells with GFP-NS3 and Flag-PPM1A; the results showed that NS3 also interacts with exogenously expressed PPM1A (Fig. 3b).

To determine which domain(s) of NS3 mediates its interaction with PPM1A, two deletion constructs were made to separately express either the protease (aa 1–180) or the helicase (aa 181–631) domain of NS3. Co-immunoprecipitation revealed that while the protease domain of NS3 interacted with PPM1A, the helicase domain did not (Fig. 3c). This suggested that the protease domain is responsible for the interaction of the full-length NS3 with PPM1A. Similarly, the protease domain of NS3 could translocate PPM1A from the nucleus to the cytoplasm (Fig. 2d). These results suggested that NS3 interacts with PPM1A through its protease domain.

### HCV infection and NS3 expression promote ubiquitin-mediated proteasomal degradation of PPM1A

**Fig. 4:**
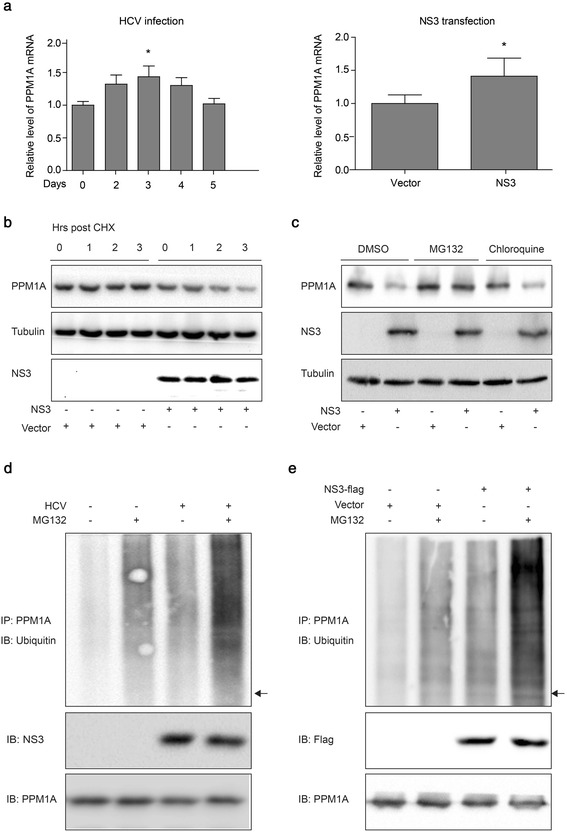
HCV infection and NS3 expression promote the degradation of PPM1A *via* the ubiquitin proteasome pathway. **a** Huh-7 cells were infected with JFH1 for 0–5 days as described in Fig. 1a (*left panel*). The cells were transfected with vector or NS3 for 36 h (*right panel*), and subsequently, the PPM1A mRNA level was determined by qRT-PCR. Data (*n* = 3) were normalized to GAPDH and presented as the fold change as compared with control cells. **b**–**c** Huh-7 cells were transfected with vector or NS3 for 36 h and subsequently treated with CHX (300 μM) for 0–3 h as indicated (**b**), vehicle (DMSO), MG132 (20 μM) or chloroquine (50 μM) for 12 h (**c**). PPM1A protein levels were detected by immunoblotting. **d**–**e** Huh-7 cells were infected with JFH1 virus (MOI ~0.5) for 72 h (**d**), transfected with Flag-vector or Flag-NS3 for 36 h (**e**), and treated with MG132 (20 μM) for 12 h. The cells were lysed and subjected to immunoprecipitation with antibody against PPM1A and analyzed by western blotting with antibodies against the indicated proteins. *Arrows* in the anti-ubquitin blots indicate the position of unmodified PPM1A proteins

Next, the mechanism underlying the decrease in PPM1A induced by HCV infection or NS3 expression was systematically explored. First, PPM1A mRNA levels were measured by qRT-PCR in Huh-7 cells infected with HCVcc for 5 days. PPM1A mRNA was upregulated in HCV-infected cells from 2 days after infection (Fig. 4a, left panel). Similarly, ectopic expression of NS3 significantly elevated the PPM1A mRNA level (Fig. 4a, right panel), likely owing to compensation. Subsequently, we assessed the protein stability of PPM1A in NS3-expressing cells after treatment with cycloheximide, a protein synthesis inhibitor. The results showed that the stability of the PPM1A protein was decreased in NS3-expressing cells (Fig. 4b). Together, these results provided strong evidence that NS3 modulates the level of PPM1A *via* posttranscriptional regulation.

Eukaryotic cells use the ubiquitin proteasome system (UPS) and autophagy lysosome pathway (ALP) as major protein degradation pathways [26]. Treatment of NS3-transfected cells with UPS inhibitor MG132 or ALP inhibitor chloroquine revealed that MG132 almost completely abolished the effect of NS3 on the PPM1A protein level (Fig. 4c). This indicated that NS3 might affect the proteasomal degradation of PPM1A.

Since ubiquitination is generally enhanced prior to proteasomal degradation [27], we examined whether HCV infection and NS3 expression enhanced the ubiquitination of PPM1A. Huh-7 cells were infected with JFH1 virus for 72 h, then treated without or with MG132 for 12 h, and immunoprecipitation experiments with an antibody specific for PPM1A were performed. Ubiquitinated PPM1A was detected using an anti-ubiquitin antibody. Apparently, the levels of ubiquitinated PPM1A were significantly higher in HCV-infected than in control cells (Fig. 4d). Moreover, NS3 overexpression also elevated the ubiquitinated PPM1A levels (Fig. 4e). Taken together, these results indicated that HCV NS3 protein downregulates PPM1A protein level by promoting its ubiquitin-dependent proteasomal degradation.

### PPM1A overexpression reverses NS3-mediated enhancement on cell invasion

**Fig. 5:**
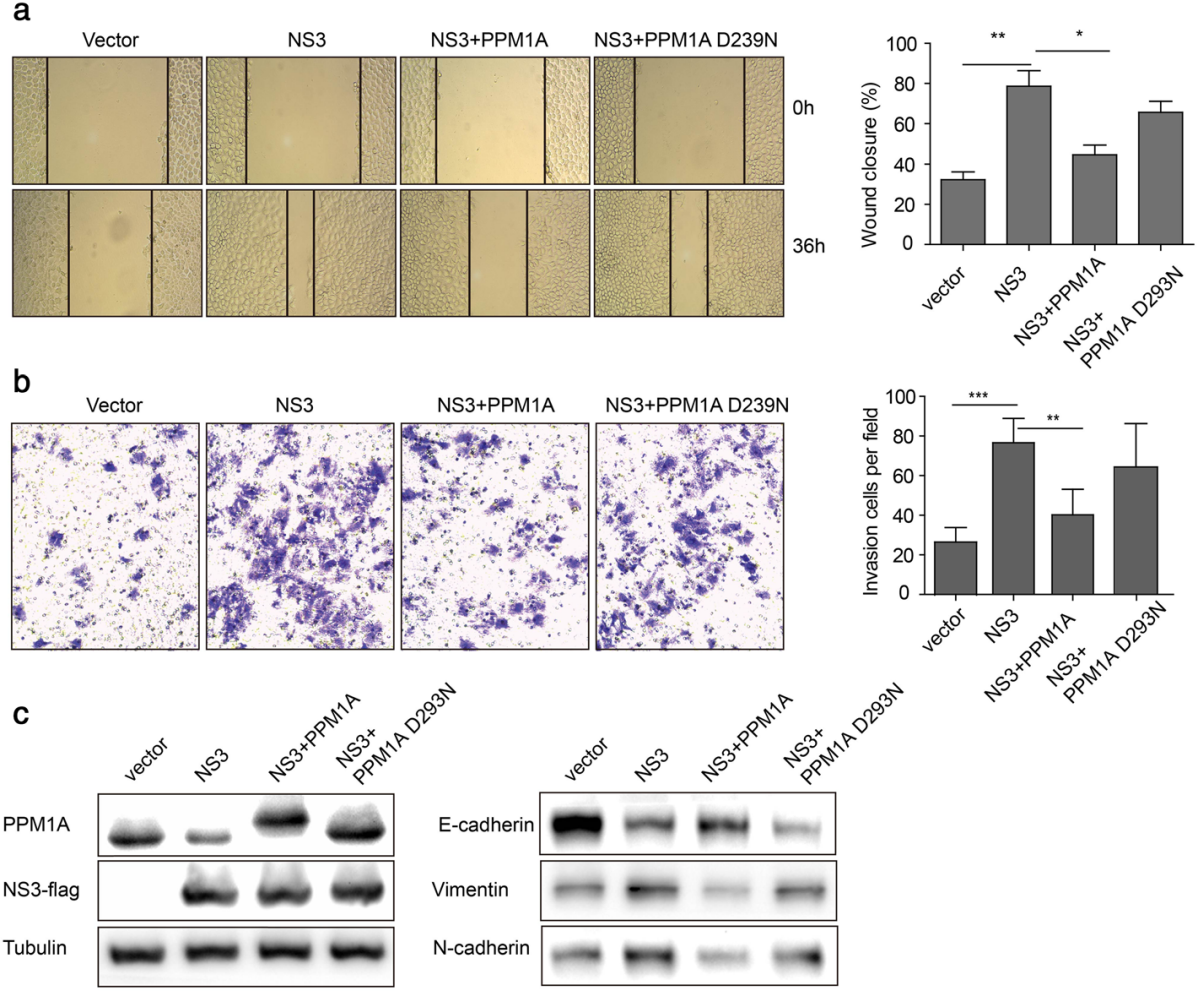
Restoration of PPM1A abundance partially reverses NS3-mediated enhancement of cell invasion. **a**–**b** After transfection with vector, NS3, NS3 and PPM1A, NS3 and PPM1A D239N, respectively, the migration and invasion capacities of Huh-7 were analyzed by wound closure (**a**) and transwell invasion (**b**) assays. **c** The protein levels of PPM1A, NS3, vimentin, E-cadherin and N-cadherin were measured by western blotting after transfection with plasmids indicated above. Data are the mean ± SEM. *P < 0.05, **P < 0.01 and ***P < 0.001 as evaluated using Student’s *t*-test

To prove that downregulation of PPM1A is involved in NS3-induced promotion of HCC metastasis, we restored the level of PPM1A in cells expressing NS3 and then measured the cell migration and invasion capacities using wound healing and transwell invasion assays. As shown in Fig. 5c, PPM1A D239N, the phosphatase-dead mutant of PPM1A, exhibited increased gel migration as compared to its wild-type counterpart. This phenomena was reported previously by Lin et al. [14], likely reflecting a structural role of this residue. In any case, transfection of 0.5 μg of PPM1A and D239N leveled PPM1A expression of NS3-transfected cells to nearly that of control cells. Moreover, wound healing and transwell invasion assays revealed that while NS3 alone enhanced cell migration and invasion, co-transfection of NS3 and PPM1A partially reversed this promotional effect, which indicates that the suppression of PPM1A by NS3 might be a novel mechanism of HCV in tumorigenesis (Fig. 5a and b).

Epithelial-mesenchymal transition (EMT) has been associated with the acquisition of motility, invasiveness, and self-renewal traits in HCC cells [28]. In this study, sole expression of the viral protein NS3 significantly increased vimentin and N-cadherin expression and decreased E-cadherin expression, while restoration of PPM1A expression nearly abrogated the EMT-promoting effect of NS3 (Fig. 5c). Moreover, we found that the phosphatase-dead mutant PPM1A D239N could not abolish the enhancing effect of NS3, which suggests that the function of PPM1A depends on its phosphatase activity (Fig. 5a and b). Together, these results suggested that the cellular invasion capacity caused by NS3 is mediated at least partially through the modulation of PPM1A abundance.

### Downregulation of PPM1A promotes the invasive capacity of Huh-7 cells in vitro, and this promotional effect is intensified by TGF-β1 stimulation

**Fig. 6:**
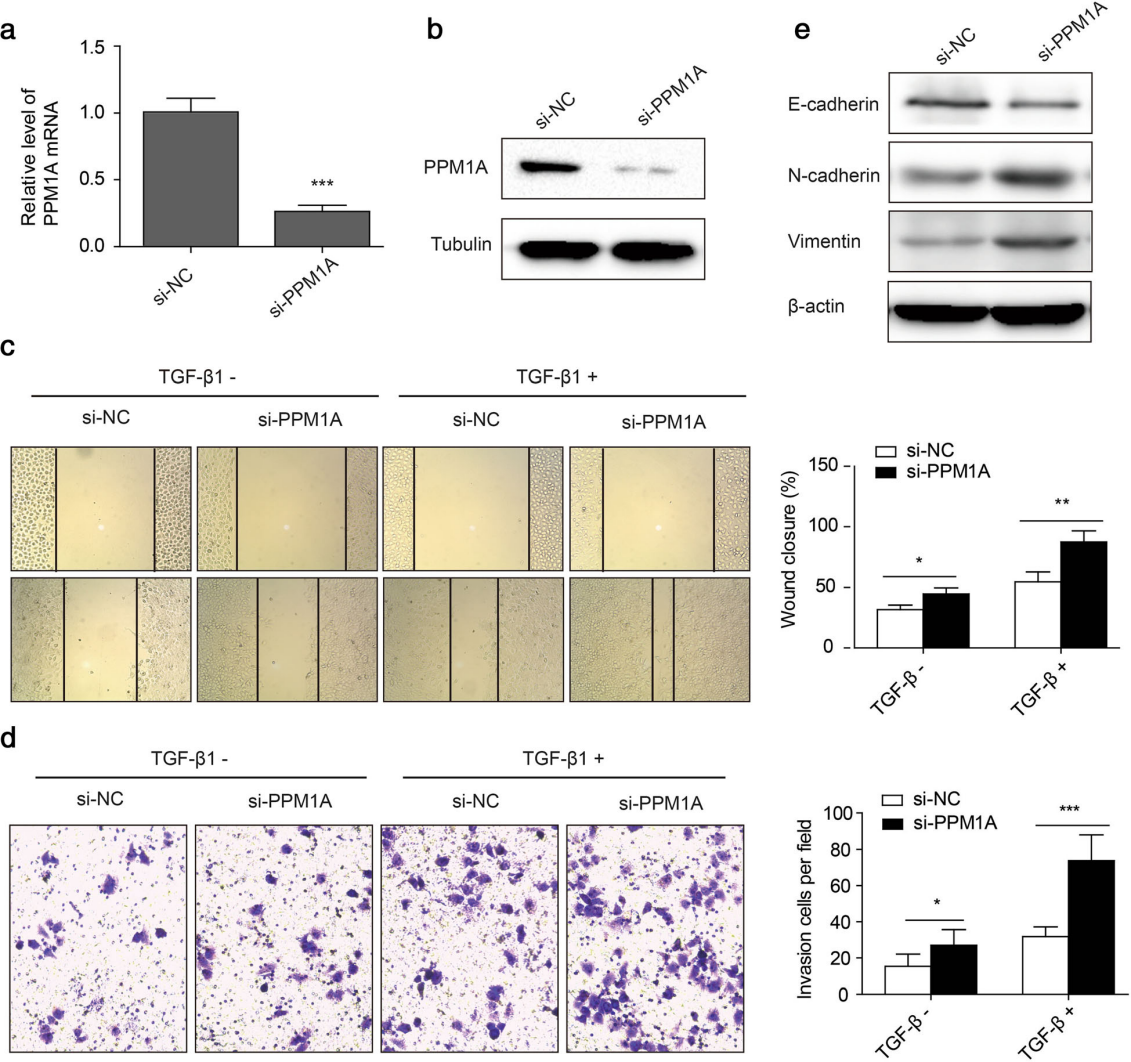
Knockdown of PPM1A promotes the invasive capacity of HCC cells, which is intensified by exogenous TGF-β1. **a**–**b** mRNA (**a**) and protein (**b**) levels of PPM1A in Huh-7 cells were assessed by qRT-PCR and western blotting, respectively. Huh-7 cells were transfected with 100 nM PPM1A siRNA (si-PPM1A) or control siRNA (si-NC) for 36 h before detection. GAPDH and tubulin served as internal controls for mRNA and protein loading, respectively. **c**–**d** Huh-7 cells were transfected with si-PPM1A or si-NC and treated with TGF-β1 (200 pM) or vehicle. Wound healing (**c**) and transwell invasion (**d**) assays were performed to analyze the migration and invasion ability of Huh-7 cells. **e** Western blot analysis for vimentin, E-cadherin, N-cadherin protein in Huh-7 cells following transfection with si-PPM1A or si-NC. Data are the mean ± SEM. *P < 0.05, **P < 0.01 and ***P < 0.001 as evaluated using Student’s *t*-test

To assess the role of PPM1A in regulating HCC cell invasiveness, we used RNA interference to examine the impact of PPM1A knockdown on Huh-7 cell migration and invasion capacities. Western blot and RT-PCR analyses revealed that the use of pools that consists of two different specific siRNAs allowed reducing endogenous PPM1A protein by over 70% relative to the level in control cells (Fig. 6a and b). Taking into account the role of PPM1A in TGF-β signaling, we further examined whether the PPM1A-mediated inhibition of the TGF-β signaling pathway would sensitize cells to TGF-β stimulation. The wound healing assay demonstrated that PPM1A-knockdown cells showed significantly faster wound closure than control RNAi cells (Fig. 6c). In the presence of TGF-β1, these differences were more remarkable. Consistent herewith, the transwell invasion assay indicated that PPM1A knockdown significantly enhanced invasion by huh-7 cells, which was augmented by TGF-β1 stimulation (Fig. 6d). These results indicated that the loss of PPM1A significantly promoted HCC cell migration and invasion in vitro, which was further intensified by TGF-β1 stimulation.

In accordance herewith, PPM1A-knockdown cells showed increased vimentin and N-cadherin expression, but decreased E-cadherin expression, when compared to control cells (Fig. 6e). These results demonstrated that the reduced expression of PPM1A resulted in a more pronounced EMT phenotype, which might contribute to the acquisition of tumor-initiating and malignancy-associated traits.

## Discussion

Increasing experimental evidence suggests that HCV contributes to HCC by directly modulating signaling pathways that promote the malignant transformation of hepatocytes. Among the HCV-encoded proteins, the core protein, NS3, NS4B, and NS5A have received much attention, since all of them possess cell-transforming potential by interacting with a number of host factors and signaling pathways when expressed in cell culture or transgenic animal models [6, 29]. NS3, a non-structural protein of HCV, contains a protease and a helicase domain and plays crucial roles in the processing of the viral polyprotein, viral RNA replication, and translation [23]. Previous studies have shown that NS3 protein modulates various signaling pathways that have transforming potential [30]. In this study, we found a new strategy adopted by NS3 to promote the invasion and metastasis of HCC cells through promoting the ubiquitination and degradation of PPM1A.

Although PPM1A was first identified approximately 20 years ago and a number of achievements with regard to revealing its functions have been made, studies on the mechanisms of PPM1A regulation have been rarely reported. In this study, we found that PPM1A was strongly downregulated and its normal nuclear localization shifted to a mainly cytoplasmic distribution following infection of cultured hepatoma cells with HCV. A screening of HCV proteins revealed that NS3 was responsible for this alteration. Our results are consistent with a mechanism in which NS3 forms a complex with PPM1A, thereby accelerating its degradation, since we showed that the stability of PPM1A is reduced in cells expressing NS3 protein. In addition, we observed a restoration of PPM1A abundance after treatment of NS3-expressing cells with proteasome inhibitors, and the amount of ubiquitin-complex PPM1A increased in HCV-infected as well as NS3-expressing cells, which suggests that NS3 modulates the abundance of PPM1A *via* the cellular normal protein degradation pathway. Significantly lower levels of PPM1A were also found in HCV-related HCC and adjacent tissues than in normal tissues. However, our data did not reveal a direct correlation between PPM1A and NS3 expression in HCC tissues because of the low level of HCV antigen expression in HCC tissues and the lack of an antibody capable of labeling the HCV protein in infected liver tissues [31].

The participation of HCV proteins in the ubiquitination and degradation of host proteins has been reported. Munakata et al. found that HCV NS5B protein traps retinoblastoma tumor-suppressor protein (pRb) in the cytoplasm and subsequently recruits E6AP to this complex, which leads to the ubiquitination and degradation of pRb [32]. NS3 can also interact with several components of the UPS, including the E3 ubiquitin ligase components DDB1 [33], LUBAC [34], and SMURF2 [12], which suggests that the utilization of the UPS by HCV proteins has become one of the strategies of HCV for promoting HCC progression and viral replication. However, the precise molecular mechanism underlying the enhancement of PPM1A ubiquitination by NS3 remains elusive and warrants further studies.

Recently, PPM1A has attracted increasing interest owing to its tumor suppressor-like activity. Lu et al. [18] found that decreased PPM1A expression enhances prostate cancer metastasis and this, at least partially, depends on its ability to inhibit NF-κB signaling. In metastatic bladder cancer, loss of PPM1A expression significantly promoted urinary bladder cancer cell motility, EMT in vitro, and metastasis in vivo, which were dependent on the TGF-β/smad signaling pathway [21]. Similarly, we found that knockdown of PPM1A significantly promoted HCC cell migration and invasion in vitro, and the promotional activity was further intensified by TGF-β1 stimulation. These results show that loss of PPM1A may play an important role in the tumorigenesis of HCC, and that it partially depends on the TGF-β/smad signaling pathway. Since multiple tumor-related pathways can be modulated by PPM1A, further studies on the exact signaling pathway and its corresponding function in HCC tumorigenesis are needed.

Lin et al. [14] found that endogenous PPM1A is primarily localized in the nucleus, where it dephosphorylates and promotes the nuclear export of TGF-β-activated Smad2/3. This study corroborated that PPM1A is normally localized in the nucleus. When NS3 was expressed, PPM1A was shuttled to the cytoplasm and it demonstrated a more diffuse pattern throughout the cells. The alteration of PPM1A subcellular localization may be partially caused by an interaction with NS3, which localizes in the cytoplasm in the presence of NS4A [25]. It is likely that NS3 interacts with and traps PPM1A in the cytoplasm where HCV completes its lifecycle, thereby accelerating its degradation. Since the effect of PPM1A on TGF-β signaling is dependent on its nuclear localization, we speculate that, in addition to a decrease in the abundance of PPM1A, its cytoplasmic redistribution might be another means to enhance TGF-β signaling. It will be of great interest to explore how the subcellular localization of PPM1A is regulated in order to control TGF-β and other signaling pathways under physiological and pathophysiological conditions.

Furthermore, we found that the rescue of PPM1A expression partially counteracted the promotional effect of NS3 on HCC cell migration and invasion, and that the rescuing efficiency depended on its protein phosphatase function as phosphatase-dead mutants of PPM1A had no effect. It is worth noting that restoration of PPM1A could only partially abrogate this promotional effect, suggesting that NS3 possesses additional carcinogenesis activity. In fact, the oncogenic properties of NS3 may involve a variety of signal-transduction pathways. In addition to modulating the TGF-β pathway by targeting SMURF2 [12], NS3 can also enhance cancer cell invasion by activating matrix metalloproteinase-9 (MMP-9) and cyclooxygenase-2 (COX-2) through the ERK/p38/NF-κB signal cascade [35], and interact with p53 to inhibit p53-dependent transcription [30]. These findings strongly suggest that the inhibition of PPM1A by NS3 may be a novel mechanism of HCV-mediated carcinogenesis.

Interestingly, during the course of the present study, Liu et al. [36] reported that the HBV X protein (HBX) increased the ubiquitination and degradation of PPM1A, which is responsible for the HBX-induced promotion of HCC carcinogenesis. This suggests that PPM1A, a key modulator of the signaling pathway, can be targeted and degraded by more than one hepatitis viral protein, and that this has become one of the strategies of the virus to promote tumor progression.

## Conclusions

We proposed a new mechanism by which HCV protein NS3 enhances cancer cell invasion and migration through promoting the ubiquitination and degradation of PPM1A. These findings not only substantiate the critical role of NS3 in developing cancer, but also suggest that strategies to increase PPM1A expression or activity in cancer cells could be explored as a therapeutic strategy for HCC prevention and treatment. However, further studies in vivo should be performed to validate the function and mechanisms of PPM1A.

## Additional file

Additional file 1: Figure S1. Huh-7 cells were infected with JFH1 (MOI = 1) for 0–5 days as described in Fig. 1a. Intracellular HCV RNA levels were then determined by RT-qPCR. Data are normalized to GAPDH. The error bars represent standard deviations of triplicates. (JPG 708 kb)

## Abbreviations

ALP: Autophagy lysosome pathway
CHX: Cycloheximide
EMT: Epithelial mesenchymal transition
HBV: Hepatitis B virus
HCC: Hepatocellular carcinoma
HCV: Hepatitis C virus
Huh-7: Human hepatoma cell
JFH1: The genotype 2a HCV strain JFH1
MAPK: Mitogen-activated protein kinase
NF-κB: Nuclear factor-κB
NS3: HCV nonstructural protein 3
p53: Tumor protein p53
PI3K: Phosphatidylinositol 3-kinase
PPM1A: Protein phosphatase magnesium-dependent 1A
pRb: Retinoblastoma tumor-suppressor protein
TGF-β: Transforming growth factor β
UPS: Ubiquitin proteasome system
