# Supplementary figures and images for: Hepatitis C virus NS3 protein enhances hepatocellular carcinoma cell invasion by promoting PPM1A ubiquitination and degradation
Source: J Exp Clin Cancer Res. 2017 Mar 10;36:42. doi: 10.1186/s13046-017-0510-8 (PMC5345236; doi:10.1186/s13046-017-0510-8)

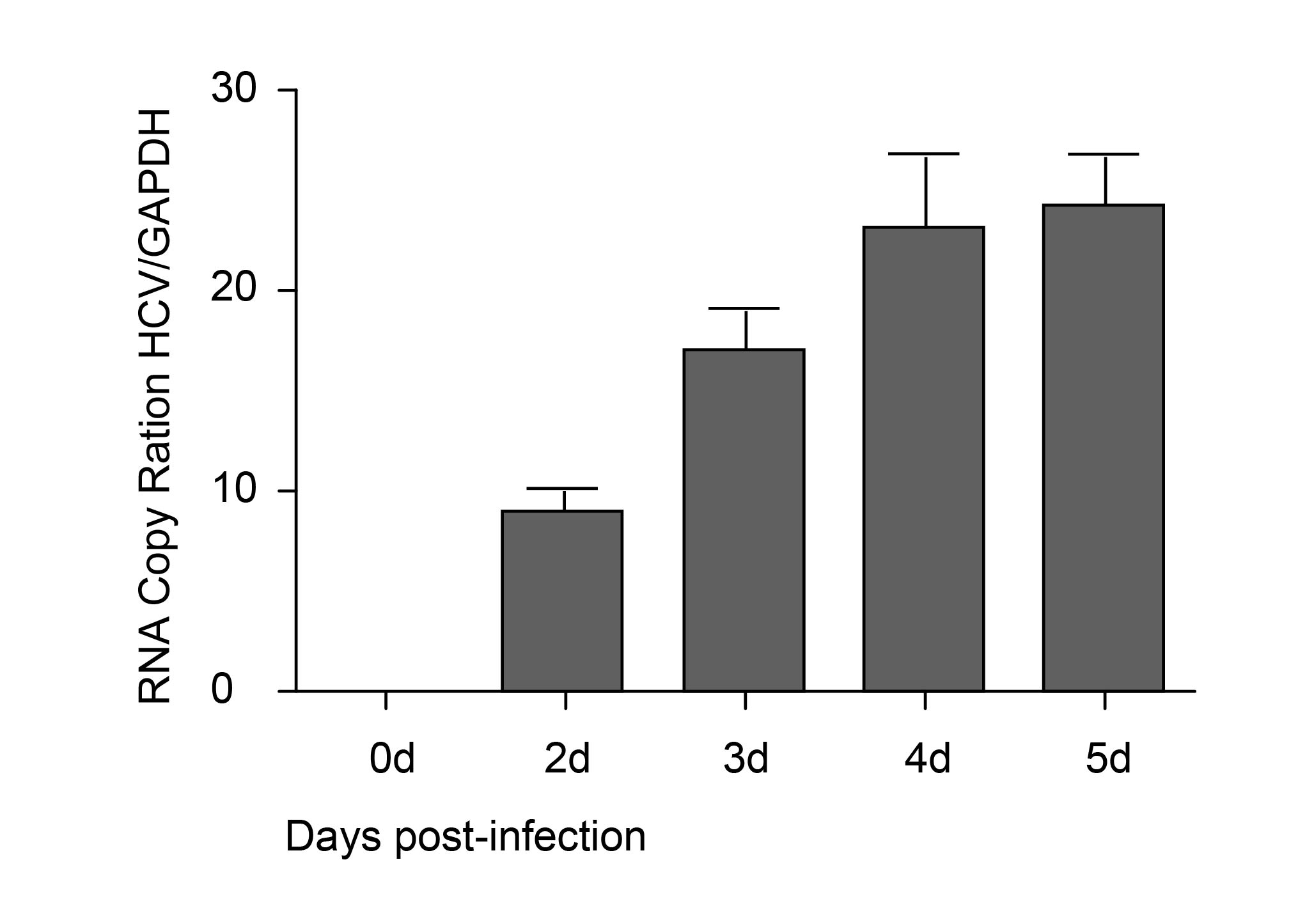

Supplement: Additional file 1: Figure S1. — Huh-7 cells were infected with JFH1 (MOI = 1) for 0–5 days as described in Fig. 1a. Intracellular HCV RNA levels were then determined by RT-qPCR. Data are normalized to GAPDH. The error bars represent standard deviations of triplicates. (JPG 708 kb) [file 13046_2017_510_MOESM1_ESM.jpg]
